# *Meteorona kishinouyei*, a new family, genus and species (Cnidaria, Cubozoa, Chirodropida) from Japanese Waters

**DOI:** 10.3897/zookeys.503.9047

**Published:** 2015-05-11

**Authors:** Sho Toshino, Hiroshi Miyake, Haruka Shibata

**Affiliations:** 1 Graduate School of Fisheries Sciences, Kitasato University, 1-15-1, Kitasato, Sagamihara, Kanagawa 252-0373, Japan

**Keywords:** Box jellyfish, Chiropsellidae, Japan, taxonomy

## Abstract

A new family, genus and species of cubozoan box jellyfish belonging to the order Chirodropida is reported from the eastern Japan. *Meteorona kishinouyei* **gen. et sp. n.** possesses the following unique morphological characters with respect to other known species in the Chirodropida: having one tentacle per scalpel-like unbranched pedalium and slightly raised unbranched gastric saccules. A comparative table of the primary diagnostic characters of genus and order in the Chirodropida is given. The order Chirodropida is redefined. The family Chiropsellidae is established. Discussion is provided on the implications for these findings on our current understanding of Cubozoan systematics.

## Introduction

The order Chirodropida currently comprises 13 species in two families, Chirodropidae and Chiropsalmidae (Gershwin 2006a; Cornelius et al. 2005; Lewis and Bentlage 2009; Bentlage 2013). Chirodropids are characterized by having multiple tentacles extending from a single branching pedalium on each corner of the swimming bell. The first identified species of Chirodropida, *Chiropsalmus quadrumanus* (Müller, 1859) was described by Müller (1859) (as *Tamoya quadrumana*). Agassiz (1862) erected the genus, Chiropsalmus Agassiz (1862) and *Tamoya quadrumana* was subsequently moved to that genus by Haeckel (1880) when he erected the order Chirodropida (as Chirodropidae) as part of Cubomedusae (formerly of the class Scyphozoa). Thiel (1936) erected the family Chiropsalmidae to replace the family Chirodropidae, when at the time it was classified as a monotypic family in the order Cubomedusae. Werner (1973) elevated Cubomedusae to the class Cubozoa that included Chirodropidae and Carybdeidae. Subsequently, Gershwin (2006a) elevated the family Chirodropidae to the order Chirodropida, and resurrected the family Chiropsalmidae. Recent molecular phylogenetic analyses and taxonomic investigations suggested that Chirodropida is a monophyletic order thought to include the paraphyletic Chiropsalmidae (Collins et al. 2006; Collins 2009; Bentlage et al. 2010).

Chirodropids have been reported from a range of tropical, sub-tropical, and mild temperature localities in the Pacific and Atlantic (Gershwin 2006a; Cornelius et al. 2005; Lewis and Bentlage 2009; Bentlage 2013). This group is infamous and well known by local fishermen, divers and bathers as dangerous box jellyfish due to their potentially lethal sting. In particular, envenomation by *Chironex yamaguchii* poses a serious problem to public health and tourism in Japan, with 100 to 200 stings reported per year, and a total of three fatalities following envenomation in Okinawa, southern Japan (Yamaguchi 1980; Okinawa Prefectural Institute for Health and Environment 1999). Currently, part of the life cycle of only one chirodropid is known: *Chironex fleckeri* (Yamaguchi and Hartwick 1980; Hartwick 1991). In an attempt to focus on countermeasures against envenomations, some workers have looked at diurnal migration and seasonal occurrence of Chirodropid medusae, and the potential habitat of polyps has been investigated using light traps (Iwanaga et al. 2005).

Five species of Japanese cubozoans have been reported, *Carybdea brevipedalia* Kishinouye, 1891, *Copula sivickisi* (Stiasny, 1926), *Tripedalia cystophora* Conant, 1897, *Morbakka virulenta* (Kishinouye, 1910), and *Chironex yamaguchii* Lewis & Bentlage, 2009 (Uchida 1929; 1970; Yamaguchi 1982; Lewis and Bentlage 2009; Bentlage et al. 2010; Bentlage and Lewis 2012; Akiyama et al. 2013) (Table 1). In this study, five specimens of an unknown cubozoan species were collected from eastern Japan. Our morphological and molecular phylogenetic analyses suggest that the cubozoan should be regarded as new family, genus and species within the order Chirodropida.

**Table 1.** List of Japanese Cubozoa. **a** Uchida (1929); **b** Uchida (1970); c Bentlage et al. 2010; **d** Akiyama et al. (2013); **e** Bentlage and Lewis (2012); **f** Yamaguchi (1982); **g** Lewis and Bentlage (2009).

Current name	Former name	Japanese name	Reference
*Carybdea brevipedalia* Kishinouye, 1891	*Carybdea rastonii*	Andon-kurage	a, b, c
*Copula sivickisi* (Stiasny, 1926)	*Carybdea sivickisi*	Himeandon–kurage	a, b, c
*Tripedalia cystophora* Conant, 1897	*Tripedalia cystophora*	Mitsuderippo–kurage	b, d
*Morbakka virulenta* (Kishinouye, 1910)	*Tamoya haplonema*	Hi–kurage	a, b, c, e
*Chironex yamaguchii* Lewis & Bentlage, 2009	*Chiropsalmus quadrigatus*	Habu–kurage	f, g

## Methods

**Figure 1.**
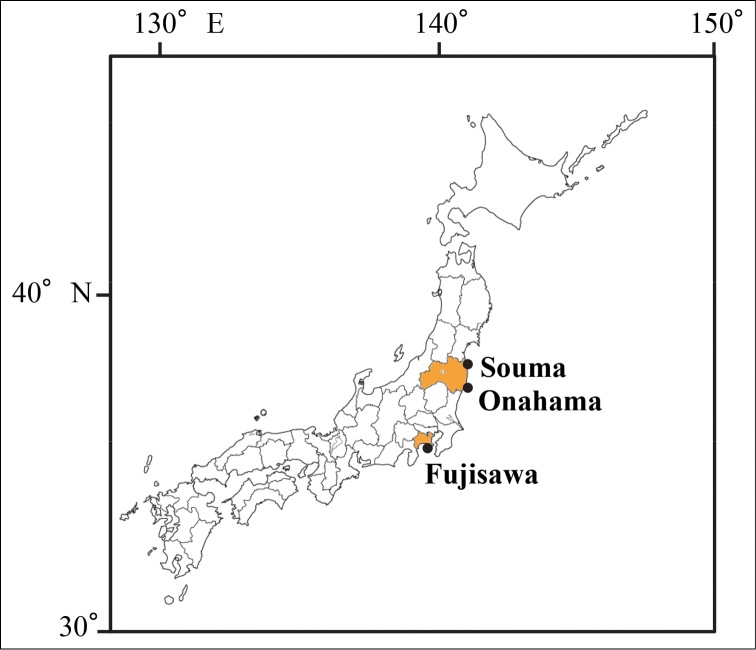
Map of the sampling sites.

Specimens were collected using a scoop, 170 mm in diameter, at Souma, Onahama and Fujisawa, eastern Japan between 24 August 2011 and 18 October 2013 (Fig. 1). The medusae ware fixed in 5% formalin in seawater, and deposited in the National Museum of Nature and Science, Tsukuba, Japan (NSMT). Prior to fixation, a tentacle subsample of each specimen was preserved in 99.5 % ethanol for DNA extraction.

**Figure 2.**
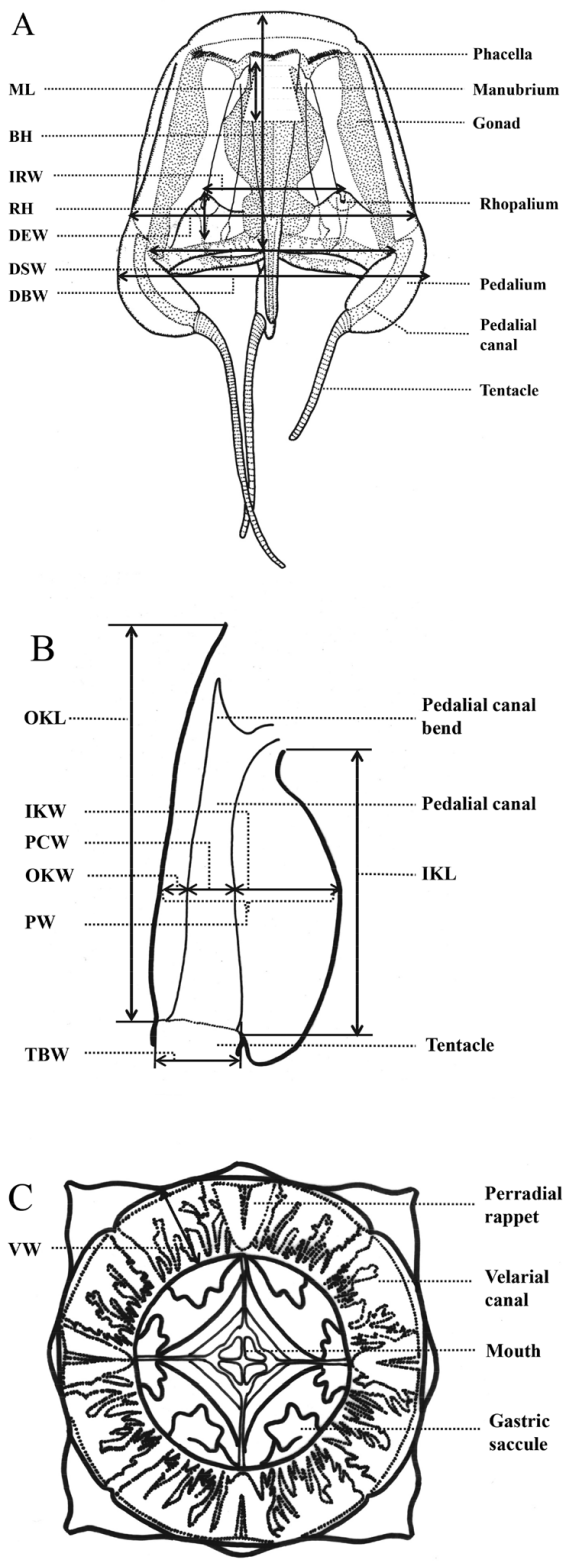
Key characters for identification and measurement of parts of the Cubozoa. **A** lateral view **B** pedalium **C** oral view. BH = Bell height; DBW = Diagonal bell width; DEW = Diagonal exumbrella width; DSW = Diagonal subumbrella width; IKL = Inner keel length; IKW = Inner keel width; IRW = Interrhopalial width; ML = manubrium length; OKL = Outer keel length; OKW = Outer keel width; PCW = Pedalial canal width; PW = Pedalial width; RH = Rhopalium height; TBW = Tentacle base width; VW = Velarial width.

Taxonomic observation and measurements were made on preserved specimens. Measurements were made with digital calipers (CD-20CPX, Mitsutoyo Corporation, Japan) to the nearest 0.01 mm. The following measurements were made according to Gershwin 2005 (Fig. 2): bell height (BH), diagonal bell width (DBW), interrhopalial width (IRW), pedalial width (PW), pedalial canal width (PCW), outer keel width (OKW), inner keel width (IKW), tentacle base width (TBW), velarial width (VW). Sex was determined by examining gonadal tissue under a light microscope. In this study, some of new measurements were added as below: Diagonal exumbrella width (DEW) and diagonal subumbrella width (DSW) were both measured across diagonal base of outer keel or inner keel of pedalia, respectively, on a flattened specimen. Outer keel length (OKL) and inner keel length (IKL) were both measured from the exumbrella lamella or subumbrellar lamella, respectively, to the tentacle insertion. Rhopalial height (RH) was measured from the base of rhopalial stalk to velarial turnover. Manubrium length (ML) was measured from the base of manubrium to mouth.

For nematocyst identification in the medusae, squashes prepared from fresh tissues were examined under a compound microscope. Nematocysts were identified according to Mariscal (1971), Östmann (2000) and Gershwin (2006b). For determination of the abundance of nematocyst types in medusae, roughly 500 nematocysts were identified, measured and counted from two specimens, NSMT-Co1569 and NSMT-Co 1571.

Near complete sequences of the nuclear 18S rDNA genes (approximately 1800 bp) were used for molecular phylogenetic analysis. Genomic DNA was extracted from the 99.5 % ethanol preserved subsampled tentacle tissue of NSMT-Co1569, NSMT-Co1571 and NSMT-Co1572 using the DNeasy Blood and Tissue Kits (Qiagen, Germany) following the manufacturers protocol. 18S rDNA was PCR amplified and sequenced using primers and protocols outlined in Collins et al. (2008). The new sequences were aligned using MEGA 6.06 with built in ClustalW. Phylogenetic analysis and pairwise distance measurements were determined using the maximum likelihood method with 1000 bootstrap replications in MEGA 6.06. All of these sequences have been deposited in DDBJ under accession numbers LC033478-LC033480 for the new species (Table 2).

**Table 2.** Taxa included in the phylogenetic analyses and GenBank accession numbers for sequences. Sequences obtained in this study are in **bold.**

Species	GenBank No.	Reference
*Alatina moseri* (Australia)	GQ849082 (as *Alatina mordens*)	Bentlage et al. 2010
*Carybdea branchi* (South Africa)	GQ849089	Bentlage et al. 2010
*Carybdea brevipedalia* (Japan)	GQ849092 (as *Carybdea mora*)	Bentlage et al. 2010
*Carybdea marsupialis*	AF358106	Bentlage et al. 2010
*Carybdea rastonii*	AF358108	Collins et al. 2002
*Carybdea xaymacana*	AF358109	Collins et al. 2002
*Carybdea xaymacana* (Panama)	GQ849090	Bentlage et al. 2010
*Carukia barnesi*	AF358107	Collins et al. 2002
*Copula sivickisi*	AF358110 (as *Carybdea sivickisi*)	Collins et al. 2002
*Gerongia rifkinae*	AF358105 (as *Darwin carybdeid*)	Collins et al. 2002
*Meteorona kishinouyei* (Fujisawa)	**LC033478**	this study
*Meteorona kishinouyei* (Onahama)	**LC033479**	this study
*Meteorona kishinouyei* (Souma)	**LC033480**	this study
*Morbakka virulenta* (Japan)	GQ849083	Bentlage et al. 2010
*Malo maxima* (Australia)	GQ849084 (as *Malo kingi*)	Bentlage et al. 2010
*Tripedalia cystophora* (Indonesia)	GQ849088	Bentlage et al. 2010
*Chironex fleckeri* (Australia)	GQ849073	Bentlage et al. 2010
*Chironex yamaguchii* (Japan)	GQ849076	Bentlage et al. 2010
*Chiropsalmus quadrumanus* (Brazil)	GQ849078	Bentlage et al. 2010
*Chiropsella bronzie*	AF358103 (as *Chiropsalmus* sp.)	Collins et al. 2002

## Results

### Phylum Cnidaria Verrill, 1865 Subphylum Medusozoa Peterson, 1979 Class Cubozoa Werner, 1973 Order Chirodropida Haeckel, 1880

#### Family Chiropsellidae fam. n.

Taxon classificationAnimaliaChirodropida

http://zoobank.org/7690E036-F8BD-4B62-A7BB-EF9DD1D6DE87

##### Family diagnosis.

Chirodropida with unbranched gastric saccules. Gastric phacellae V-shaped or horseshoe shaped. Sensory niches U-shaped, with medial flap on upper rhopaliar scale. Pedalial canal bend slight volcano or knee-shaped. Pedalia four, branched with 5 to 11 tentacles or unbranched with one tentacle.

##### Type genus.

*Chiropsella* Gershwin, 2006.

*Chiropsella bronzie* Gershwin, 2006: 25–36, pl. 4–6.

*Chiropsella bart* Gershwin & Alderslade, 2006: 15–21, figs 1–4.

*Chiropsella rudloei* Bentlage, 2013: 1–7, figs 1–3.

#### Genus Meteorona gen. n.

Taxon classificationAnimaliaChirodropidaChiropsellidae

http://zoobank.org/E3AA7CD7-8DE7-4BEE-9151-2D6EAAD54793

##### Genus diagnosis.

Chiropsellidae with smooth, unbranched, slightly raised gastric saccules. Gonads leaf-shaped. Gastric phacellae horseshoe-shaped. Sensory niches U-shaped, with medial tongue-shape flap on upper rhopaliar scale. Pedalia four, unbranched, scalpel-like with one tentacle per pedalium.

##### Type species.

*Meteorona kishinouyei* sp. n. here designated.

##### Etymology.

The genus name comes from the meteor-like appearance of the jellyfish shooting through the sea while swimming. The name *Meteorona* is taken from the Latin ‘Meteoron’, with the suffix -a. Gender is feminine.

#### Meteorona kishinouyei sp. n.

Taxon classificationAnimaliaChirodropidaChiropsellidae

http://zoobank.org/02513932-6CFC-4A34-87AD-863677521769

New Japanese name: Ryusei-kurage

Figures 3 , 4 , 5 , 6 , 7 , 8 , 9 , 10 , 11 , 12

##### Material examined.

Holotype: NSMT-Co1572. Eastern Japan, Fukushima Prefecture, Souma, Matsukawa-ura, 37°48'39.3"N, 140°58'3.3"E, 14 October 2013, collector: Ko Tomikawa, one adult male. Paratypes. NSMT-Co1568, 1569, 1570. Eastern Japan, Kanagawa Prefecture, Fujisawa, Enoshima, Shonan Port, 35°18'4.75"N, 139°28'61.0"E, 23 August 2011, collector: Haruka Shibata, one adult female. NSMT-Co1571. Eastern Japan, Fukushima Prefecture, Iwaki, Onahama Port, 36°56'29.1"N, 140°54'.32.9"E, 18 October 2013, collector: Shun Ishikawa, one adult female.

##### Description.

**Figure 3.**
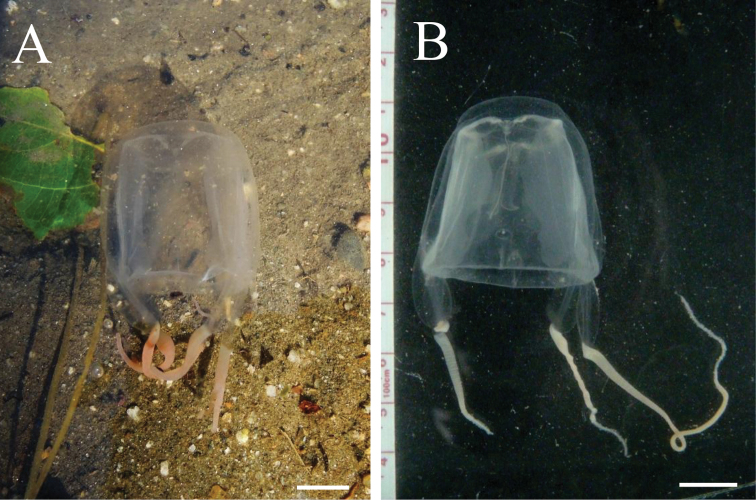
*Meteorona kishinouyei* sp. n., holotype, live, lateral view. **A** in situ, photo courtesy of Ko Tomikawa **B** in laboratory, photo courtesy of Yusuke Kondo. All scale bars represent 1 cm.

**Figure 4.**
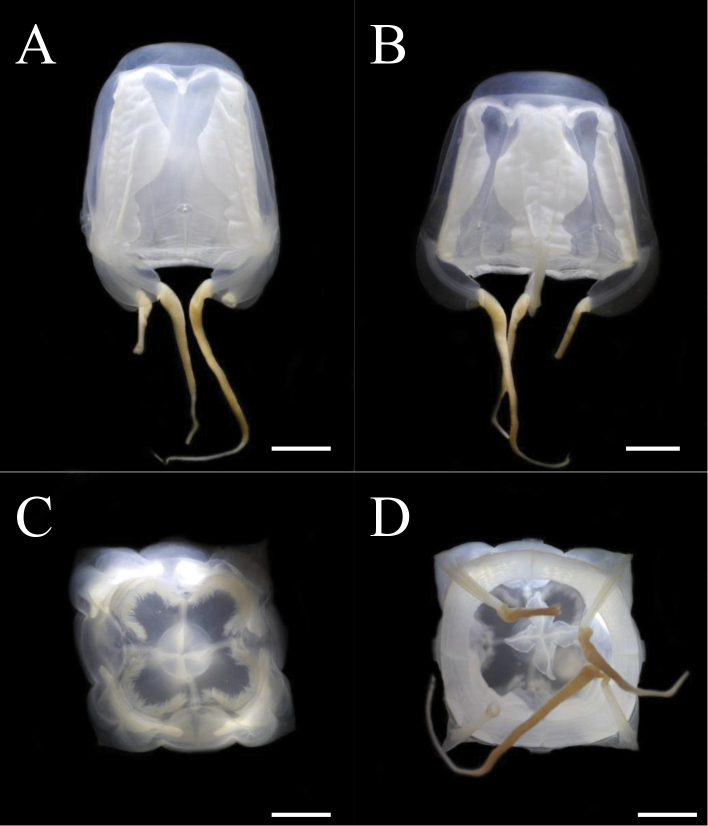
*Meteorona kishinouyei* sp. n., holotype. **A, B** lateral view **C** apical view **D** oral view. All scale bars represent 1 cm.

**Figure 5.**
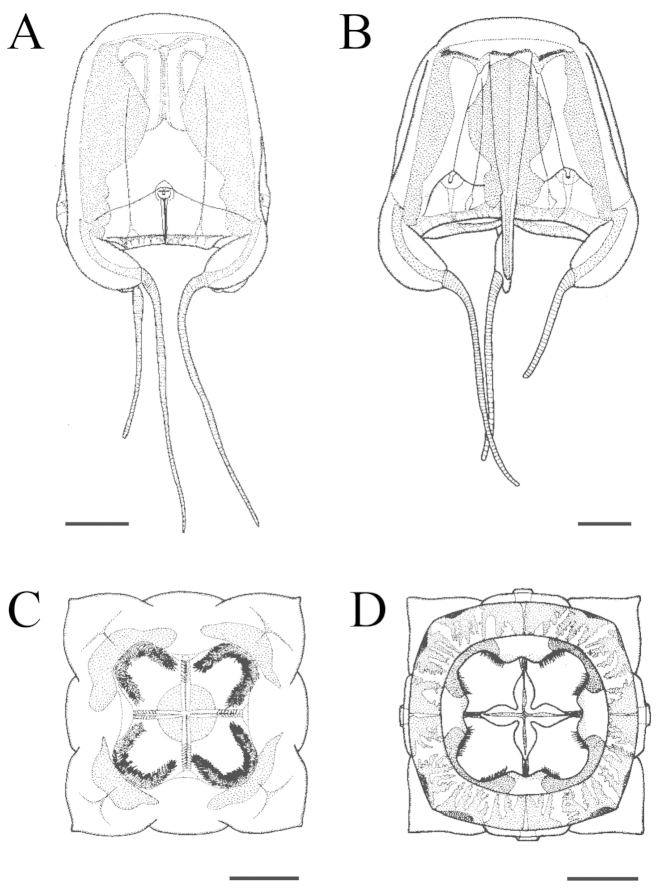
Illustration of *Meteorona kishinouyei* sp. n., holotype. **A, B** lateral view **C** apical view **D** oral view. All scale bars represent 1 cm.

**Figure 6.**
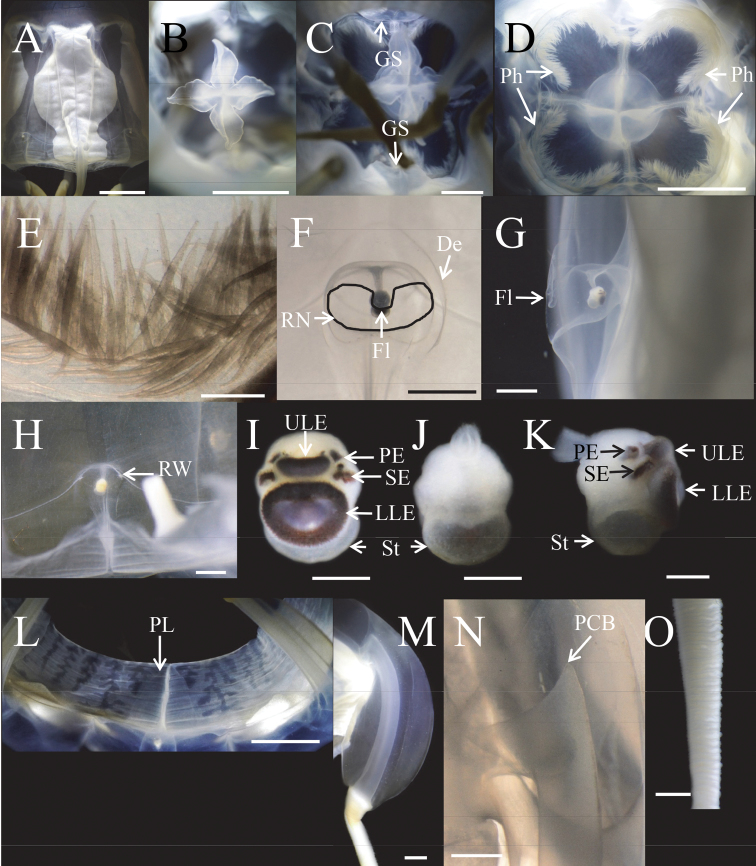
*Meteorona kishinouyei* sp. n., holotype. **A** gonad **B** mouth oral lips **C** gastric saccules **D** phacellae **E** gastric filaments **F** rhopaliar niche ostia, front view **G** rhopaliar niche ostia, side view **H** rhopaliar window **I** rhopalium, front view **J** rhopalium, rear view **K** rhopalium, side view **L** velarium; **M** pedalium **N** pedalial canal bend **O** tentacle. De: Depression; Fl: flap; GS: gastric saccule; LLE: lower lens eye; PCB: pedalial canal bend; PE: pit eye; Ph: phacella; PL: perradial lappet; RN: rhopaliar niche ostia; RW: rhopaliar window; SE: slit eye; St: statolith; ULE: upper lens eye. Scale bars: 1 cm (**A–E, L**), 5 mm (**C**), 2 mm (**F–H, M–O**), 1 mm (**E**), 0.5 mm (**I–K**).

Adult medusae with smooth exumbrella, with evenly thick mesoglea of rigid consistency (Fig. 3, Fig. 4, Fig. 5). BH about 35 mm and DBW about 50 mm (Table 3). Interradius thickened throughout bell height, with deep median furrow spanning height of bell. Adradial furrows spanning lower two thirds of bell. Coronal indentation shallow just below apex. Exumbrella lacking nematocyst warts or freckles. Gonads attached along entire length of interradial septa, leaf-shaped, not overlapping along the interradius (Fig. 6A, 7A). Manubrium length about 40% of bell height. Manubrium cruciform with four narrow, lanceolate lips (Fig. 6B).

**Figure 7.**
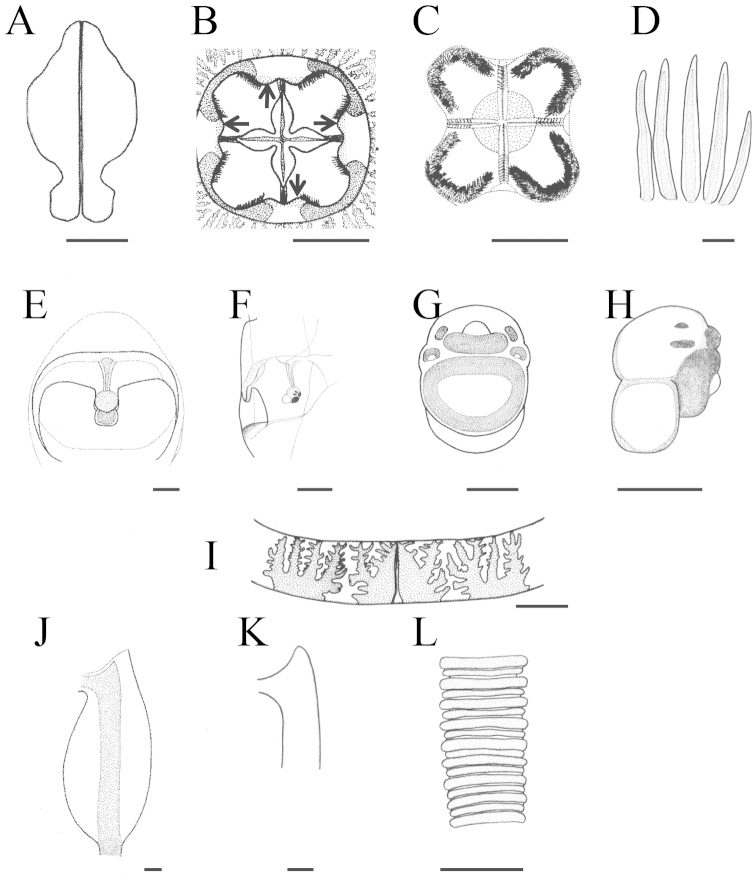
Illustration of *Meteorona kishinouyei* sp. n., holotype. **A** gonad **B** gastric saccule **C** phacellae **D** gastric filaments **E** rhopaliar niche ostia and opening, front view **F** rhopaliar niche ostia and opening, side view **G** rhopalium, front view **H** rhopalium, side view **I** velarium **J** pedalium **K** pedalial canal bend **L** tentacle. Allows indicate gastric saccule. Scale bars: 1 cm (**A–C, I**), 2 mm (**D, J–L**), 1 mm (**E–F, H**), 0.5 mm (**G**).

**Table 3.** Size (mm) of *Meteorona kishinouyei*. *: The holotype. Nos. Co1568-1571 are paratypes. BH = bell height; DBW = diagonal bell width; DEW = diagonal exumbrella width; DSW = diagonal subumbrella width; ML = manubrium length; IKL = inner keel length; IKW = inner keel width; IRW = interrhopalial width; OKL = outer keel length; OKW = outer keel width; RH = rhopalium height; PCW = pedalial canal width; PW = pedalial width; TBW = tentacle base width; VW = velarial width. All bars represent unavailable due to dissection.

Specimen No.	BH	DBW	DEW	DSW	IRW	PW	PCW	OKW
NSMT-Co1568	34,3	49,5	44,0	41,3	22,6	7,3	1,5	2,8
NSMT-Co1569	28,7	41,7	–	–	19,6	6,8	1,2	2,6
NSMT-Co1570	15,3	19,6	18,9	15,5	9,2	3,7	1,0	1,1
NSMT-Co1571	23,7	28,3	25,5	22,6	15,4	6,7	1,5	2,9
NSMT-Co1572*	34,6	46,5	42,9	39,6	22,0	6,9	1,8	3,1
**Specimen No.**	**IKW**	**OKL**	**IKL**	**TBW**	**RH**	**VW**	**ML**	**SEX**
NSMT-Co1568	3,0	17,2	11,2	2,6	5,6	5,5	15,2	Male
NSMT-Co1569	3,2	13,7	7,1	2,1	4,4	5,4	11,9	Female
NSMT-Co1570	1,3	7,5	4,9	0,9	2,3	2,8	3,6	Female
NSMT-Co1571	2,3	16,4	11,1	2,0	4,5	4,3	10,0	Female
NSMT-Co1572*	2,1	17,7	11,1	2,7	5,1	6,0	12,5	Female

Gastric saccules unbranched, slightly raised and opaque (Fig. 5D, 6C, 7B). Gastric phacellae horseshoe-shaped in each corner of stomach (Fig. 6D, 7C). Gastric cirri simple and unbranched (Fig. 6E, 7D). Sensory niches four, perradial, U-shaped, with one shallow covering scale above and no lower scale, upper scale with central tongue-shaped flap partially shielding rhopalium, located approximately 1/6 of bell height from velarial turnover (Fig. 6F, G, 7E, F). The rhopaliar niche located in a triangular shaped depression of exumbrella (Fig. 6F, 7E). Subumbrellar rhopalial windows flat (Fig. 6H). Each of the four rhopalia bearing a set of six eyes, with the two median possessing prominent lenses and the four lateral ones adjacent to the lens eyes being pigment pits and slit (Fig. 6I–K, Fig. 7G, H). A single broad bean shaped statolith located behind each complex eye (Fig. 6I–K). Velarial canals one per octant, dendritic, with only the tips defined along the velarial margin (Fig. 6L, Fig. 7I). Frenulum a single solid, gelatinous structure, extending to near velarial margin (Fig. 6L). Velarial width about 20% of DSW. Pedalia scalpel-like, each bearing one tentacle (Fig. 6M, Fig. 7J). Pedalia about half of bell height, inner keel rounded, about two times the width of pedalial canal. Outer keel width approximately twice that of pedalial canal, inner keel width and outer keel width almost equal. Proximal pedalial canal bend slight volcano-shaped (Fig. 6N, Fig. 7K). Tentacles four, with one per pedalium, base width up to 2.7 mm thick, round in cross section, with nematocyst rings alternate thick and thin in preserved specimens (Fig. 6O, 7L). Color and length of tentacles in living specimens was light brownish (Fig. 3A, B).

**Figure 8.**
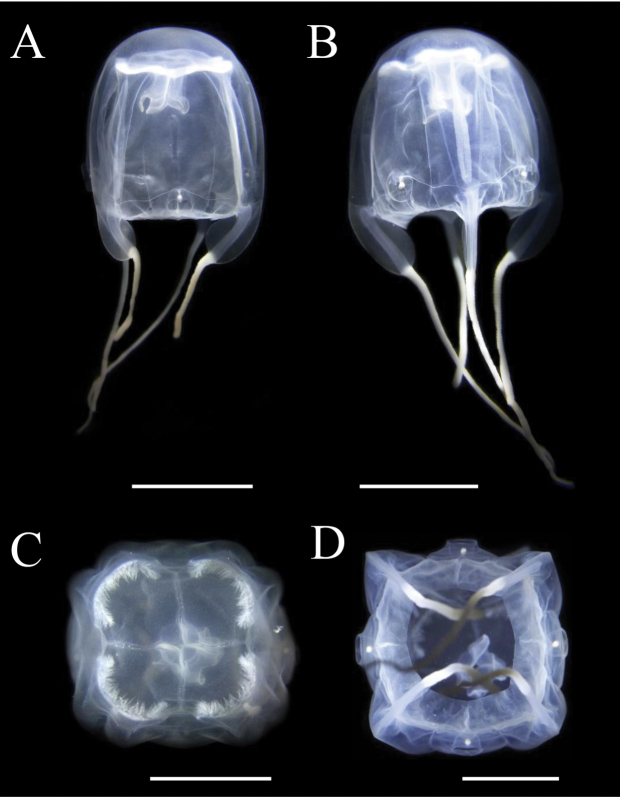
*Meteorona kishinouyei* sp. n., young medusa, paratype NSMT-Co1570. **A, B** lateral view **C** apical view **D** oral view. All bars represent 1 cm.

**Figure 9.**
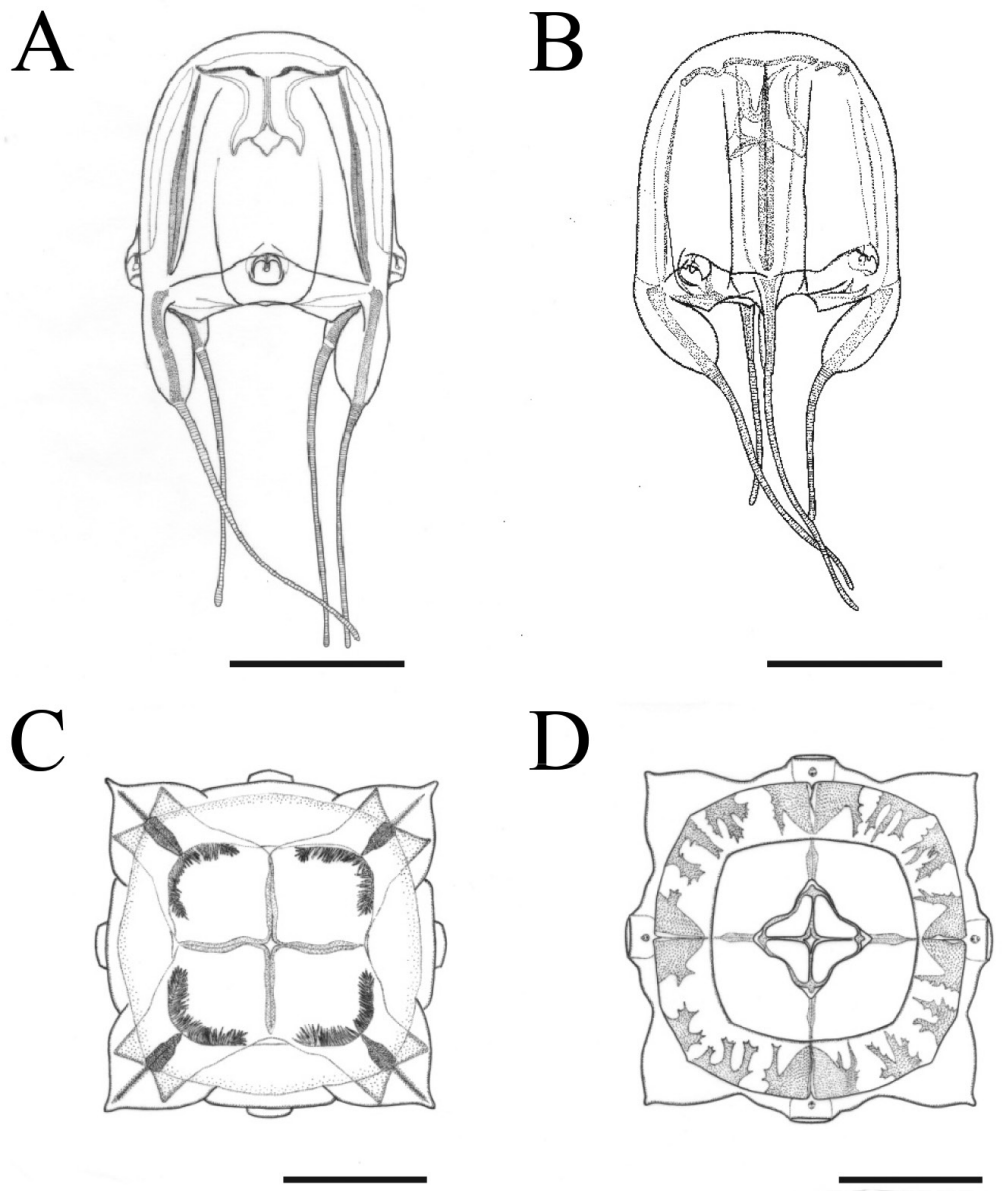
Illustration of *Meteorona kishinouyei* sp. n., young medusa, paratype NSMT-Co1570. **A, B** lateral view **C** apical view **D** oral view. All bars represent 1 cm.

**Figure 10.**
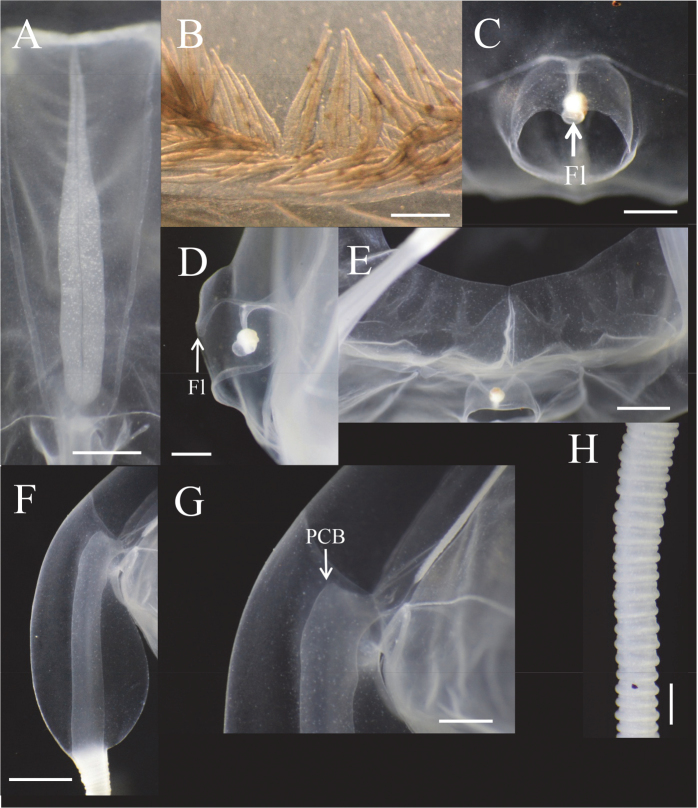
*Meteorona kishinouyei* sp. n., young medusa, paratype NSMT-Co1570. **A** gonad **B** phacella **C** rhopaliar niche ostia, front view **D** rhopaliar niche ostia, side view **E** velarium **F** pedalium **G** pedalial canal bend **H** tentacle. Fl: Flap; PCB: Pedalial canal bend Scale bars: 2 mm (**A, E–F**), 1 mm (**C–D, G–H**), 0.5 mm (**B**).

The smallest young medusa (NSMT-Co 1570) had a BH of about 15 mm, DBW of about 20 mm (Fig. 8, 9). Mesoglea was thin and softer than that of adults. Adradial furrows spanning half of the bell height (Fig. 8A, 9A). Interradial furrows spanning the entire height of the bell (Fig. 8B, 9B). Coronal indentation shallow and exumbrella nematocyst freckles absent. Gonads attached along entire length of interradial septa, leaf-shaped, not overlapping along the interradius (Fig. 10A). Manubrium length about 20% of bell height. Gastric saccules not developed. Gastric phacellae four-leaf clover shaped in each corner of stomach (Figs 8C, 9C, 10B). Depression of exumbrella not developed. Upper medial rhopaliar scale flap shorter than in adults (Fig. 10C, D). Velarial canals one per octant, dendritic but with minor branching (Fig. 10E). Pedalium scalpel-like (Fig. 10F). Volcano-shaped pedalial canal bend smoother than in adults (Fig. 10G). Tentacle with nematocyst rings alternate thick and thin in preserved specimens (Fig. 10H).

**Figure 11.**
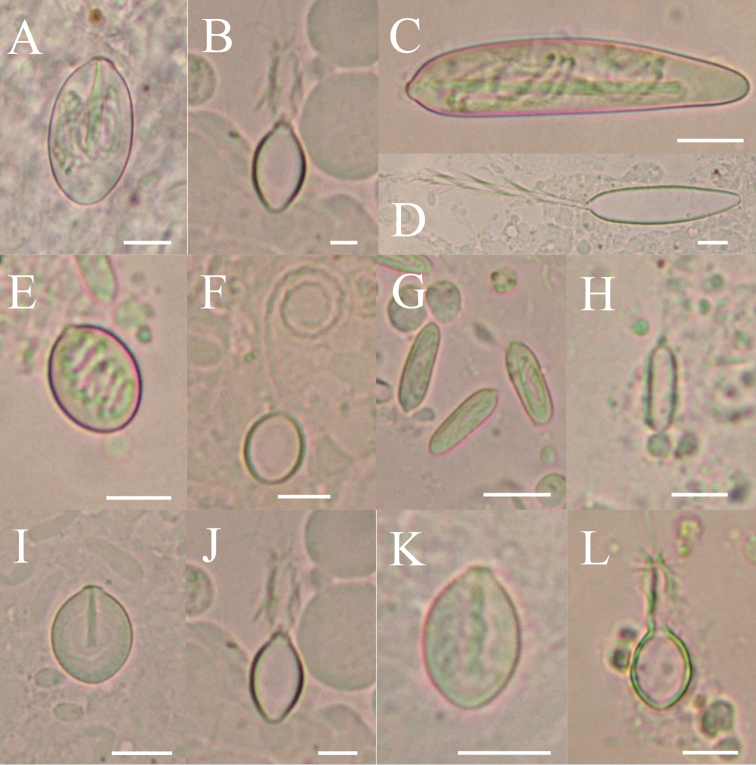
Nematocysts of *Meteorona kishinouyei*, paratype NSMT-Co1571. **A, B** Large microbasic *p*-rhopaloids. Intact (**A**), discharged (**B**) **C, D** Banana-shaped *p*-mastigophores. Intact (**C**), discharged (**D**) **E, F** Small oval beehive isorhizas. Intact (**E**), discharged (**F**) **G, H** Rod-shaped isorhizas. Intact (**G**), discharged (**H**) **I, J** Small sub-spherical *p*-rhopaloids. Intact (**I**), discharged (**J**) **K, L** Tiny microbasic euryteles. Intact (**K**), discharged (**L**). All bars represent 10 µm.

Cnidome. Six different nematocyst types identified and measured in the paratype specimen (NSMT-Co1571) (Table 4, Fig. 11). Tentacle: Large microbasic *p*-rhopaloids, banana-shaped *p*-mastigophores, small oval beehive isorhizas, rod-shaped isorhizas, small sub-spherical *p*-rhopaloids. Manubrium: Tiny microbasic euryteles, small sub-spherical *p*-rhopaloids. Phacellae: Tiny microbasic euryteles, small sub-spherical *p*-rhopaloids. Exumbrella lacking nematocysts.

**Table 4.** Cnidomes of *Meteorona kishinouyei*, paratype (NSMT-Co1571). D, L represent capsule diameter and length, respectively, in µm.

Part	Type		Min	Max	Mean	SD	N
Tentacle	banana-shaped microbasic *p*-mastigophore	D	11,58	15,49	13,62	1,06	21
		L	58,22	67,85	62,60	2,54	21
	large microbasic *p*-rhopaloid	D	15,64	20,61	17,64	1,21	30
		L	26,05	33,02	29,27	1,65	30
	small sub-spherical *p*-rhopaloid	D	11,63	15,49	13,57	1,08	25
		L	15,09	19,96	17,37	1,31	25
	small oval beehive isorhiza	D	9,77	11,86	10,67	0,56	30
		L	12,36	15,12	13,38	0,72	30
	rod-shaped isorhiza	D	3,38	5,92	4,56	0,62	30
		L	9,92	18,31	15,11	1,45	30
Exumbrella	N/A	D	–	–	–	–	–
		L	–	–	–	–	–
Manubrium	small sub-spherical *p*-rhopaloid	D	12,45	16,67	14,50	0,97	30
		L	16,15	19,35	17,79	0,90	30
	tiny microbasic eurytele	D	9,80	12,19	11,06	0,61	30
		L	14,29	18,69	16,22	0,98	30
Phacella	small sub-spherical *p*-rhopaloid	D	11,73	14,39	13,27	0,63	30
		L	14,03	18,42	16,37	1,00	30
	tiny microbasic eurytele	D	9,63	12,49	10,82	0,83	15
		L	12,56	19,79	15,82	1,91	15

##### Molecular phylogenetics.

**Figure 12.**
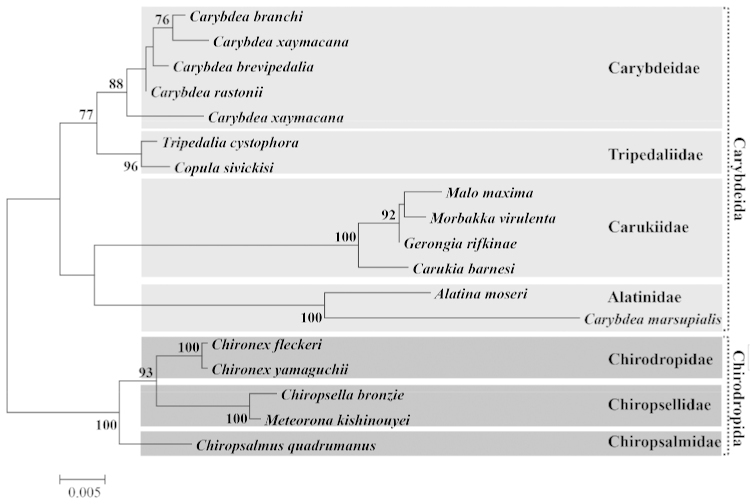
Maximum-likelihood tree for 18 cubozoan taxa based on the nuclear 18S rDNA data set. Scale bars indicate branch length in substitutions per site. Nodal support values are presented as the ML bootstrap value; only values >50% are shown.

In the resulting ML tree (Fig. 12), three major monophyletic clades were formed in the order Chirodropida: 1) Chirodropidae (*Chironex fleckeri* and *Chironex yamaguchii*); 2) Chiropsalmidae (*Chiropsalmus quadrumanus*); 3) A third group (*Meteorona kishinouyei* and *Chiropsella bronzie*). The monophyly of the third group was evident in the 18S phylogenetic tree with high bootstrap values, well supporting the validity of the new family.

##### Habitat and ecology.

Holotype specimen of *Meteorona kishinouyei* was collected near shore in shallow waters of the Matsukawa-ura lagoon, Fukushima prefecture, eastern Japan. Until March 2011, the north part of the lagoon extended to the sea via a man-made channel. However, the channel was destroyed and considerable sea water flowed into the lagoon as a result of the 2011 Tohoku earthquake and tsunami. *Meteorona kishinouyei* medusae may have been brought into the lagoon on the high waves. Moreover, *Meteorona kishinouyei* may be a neritic species. Young medusae appeared during August, while adult medusae appeared between August and November. *Carybdea brevipedalia* was collected together with *Meteorona kishinouyei* along at Onahama port and Shonan at the water surface in a shallow area (water depth 3–10 m) port during daytime. Polyps of *Meteorona kishinouyei* may metamorphose into young medusae during summer. However, the polyp stage and life history are unknown.

##### Etymology.

The species is named *kishinouyei* (noun in apposition) to honor Professor Kamakichi Kishinouye, zoologist and fishery scientist. Kishinouye’s meticulous studies and observations have led to many important contributions in the description of cubozoan zoology

##### Differential diagnosis.

A comparison of key features of the species of Chirodropida is presented in Table 5. *Meteorona kishinouyei* can be distinguished from all other chirodropid species by shape of pedalium, gastric saccules and rhopaliar niche. All of the species in the order Chirodropida have branching pedalia, each bearing multiple tentacles. *Meteorona kishinouyei* has one tentacle per unbranching pedalium like species of the order Carybdeida. Shape of gastric saccules are cock’s-comb shaped/grape-cluster-like (*Chironex*), elongate, tapered, with numerous axial processes, or absent (*Chirodropus*) in Chirodropidae, finger-like in Chiropsalmidae, spherical in *Chiropsella*. *Meteorona kishinouyei* were slightly raised. All chirodropids have sensory niches located in a triangular shaped depression of the exumbrella. However, only *Meteorona kishonouyei* and *Chiropsella* have a central flap hanging down from the upper scale of the rhopaliar niche. Exumbrella nematocysts are lacking in all chirodropid species expect *Chiropsalmus*.

**Table 5.** Morphology of chirodropida medusae in previous and the present studies. All bars represent lacking data.

Family	Genus	Nematocysts on exumbrella	Gastric saccule shape	Gastric phacella shape	Rhopaliar covering scale	Number of tentacles	Pedalial branching pattern	Pedalial canal	Pedalial canal bend
Chiropsellidae fam. n.	*Meteorona* gen. n.	–	Slightly raised	Horseshoe shaped	Tongue	1	Unbranching	Undivided	Slight volcano
	*Chiropsella*	–	Solid and spherical; sessile	V-shaped	Cigar/Banana/Tongue/Squarish to rounded	5–11	Usually but always opposite	Divided	Knee-shaped/ Slight volcano
				Horseshoe shaped					
Chirodropidae	*Chirodropus*	–	Elongate, tapered, with numerous axial processes, or absent	V-shaped	–	9–21	Bilateral with reduced ‘palm’	Unknown	Spike
	*Chirodectes*	–	–	Arranged vertically along stomach wall	–	9–11	Bilateral with reduced ‘palm’	Unknown	Spike
	*Chironex*	–	cock’s-comb shaped/grape-cluster-like	V-shaped	–	7–15	Alternate	Divided	Spike/volcano
Chiropsalmidae	*Chiropsalmus*	Fine warts	Finger-like and short	Horseshoe shaped	–	2–9	Opposite	Undivided	Slight volcano
	*Chiropsoides*	–	Finger-like and long	V-shaped	–	4–11	Unilateral	Undivided	Spike

## Discussion and conclusions

*Meteorona kishinouyei* is most likely to be confused with the carybdeid *Carybdea brevipedalia* from eastern and northern Japan. Kishinouye (1891) described two cubozoan species, *Carybdea brevipedalia* and *Carybdea latigenitalia*, collected from Shima (Mie prefecture, western Japan) and Hitachi (Ibaraki prefecture, eastern Japan) respectively. Box jellyfish with similar descriptions have been reported as *Carybdea rastoni* or *Carybdea mora* (Kramp 1961; Gershwin and Gibbons 2009). However, recent molecular phylogenetic analyses and taxonomic investigations suggest that *Carybdea rastoni* reports from Japan should be regarded as *Carybdea brevipedalia* (Bentlage et al. 2010; Bentlage and Lewis 2012). However, the account and drawing of *Carybdea latigenitalia* in the original description agree well with *Meteorona kishinouyei*: dendritic velarial canals, well developed mouth lips, half-moon shaped phacellae, very wide gonads and one tentacle per unbranching long pedalium (Kishinouye 1891). However, Uchida (1929) suggest that *Carybdea latigenitalia* should be regarded as *Carybdea rastonii*. Unfortunately, it is likely that the material investigated for the original description of *Carybdea latigenitalia* was lost. Additionally, as the name has not been in common usage, and it no longer fits the description of a species of the genus *Carybdea*, there is no justification for/obligation to resurrect the species name (ICZN 1999).

Two families, Chirodropidae and Chiropsalmidae are currently classified in the order Chirodropida (Gershwin 2006a) defined by Gershwin (2006a) as: Cubozoa with branched pedalia bearing numerous tentacles; with or without gastric saccules. However, *Meteorona kishinouyei* has one tentacle per unbranched pedalia and leaf-like gonads. We therefore propose emending the order Chirodropida as follows:

### Order Chirodropida Haeckel, 1880, sens. emend.

**Diagnosis.** Cubozoa with or without gastric saccules; with branched or unbranched pedalium; with a triangular shaped depression of exumbrella surrounded rhopaliar niche; with a triangular shaped perradial lappet and highly divergent branching velarial canals.

Based on our maximum likelihood study, *Meteorona kishinouyei* and *Chiropsella bronzie* appear to be the closest relatives in the currently understood cubozoan phylogenetic relationships. Moreover, *Meteorona* and *Chiropsella* have some common morphological characters such as the unbranched gastric saccule, rhopaliar flap and slight volcano-shaped pedalial canal bend. However, the shape of pedalium and number of tentacles differs between the two species.

*Chiropsella* has been classified as a genus in the family Chiropsalmidae (Gershwin 2006a, b, Gershwin and Alderslade 2006, Bentlage 2013). Family Chiropsalmidae defined as follows by Gershwin (2006a): Chirodropida with smooth, unbranched, finger-like gastric saccules, lacking gastric filaments. However, *Chiropsella* has gastric filaments in its stomach (Gershwin 2006a, b, Gershwin and Alderslade 2006, Bentlage 2013). Therefore, the establishment of the family Chiropsellidae is proposed that includs *Chiropsella* and *Meteorona*.

## Supplementary Material

XML Treatment for Chiropsellidae

XML Treatment for Meteorona

XML Treatment for Meteorona kishinouyei
